# Evidence for use of damage control surgery and damage control interventions in civilian trauma patients: a systematic review

**DOI:** 10.1186/s13017-021-00352-5

**Published:** 2021-03-11

**Authors:** Derek J. Roberts, Niklas Bobrovitz, David A. Zygun, Andrew W. Kirkpatrick, Chad G. Ball, Peter D. Faris, Henry T. Stelfox, for the Indications for Trauma Damage Control Surgery International Study Group, Karim Brohi, Scott D’Amours, Timothy C. Fabian, Kenji Inaba, Ari K. Leppäniemi, Ernest E. Moore, Pradeep H. Navsaria, Andrew J. Nicol, Neil Parry

**Affiliations:** 1 Division of Vascular and Endovascular Surgery, University of Ottawa, Ottawa, ON Canada; 2 Clinical Epidemiology Program, Ottawa Hospital Research Institute, The Ottawa Hospital, Ottawa, ON Canada; 3 Nuffield Department of Primary Care Health Sciences, University of Oxford, Oxford, UK; 4 Division of Critical Care Medicine, University of Alberta, Edmonton, AB Canada; 5 Department of Surgery, University of Calgary, Calgary, AB Canada; 6 Department of Critical Care Medicine, University of Calgary and Alberta Health Services, Calgary, AB Canada; 7 The Regional Trauma Program, University of Calgary and the Foothills Medical Center, Calgary, AB Canada; 8 Department of Oncology, University of Calgary and the Foothills Medical Centre, Calgary, AB Canada; 9 Alberta Health Sciences Research–Research Analytics, University of Calgary and the Foothills Medical Centre, Calgary, AB Canada; 10 O’Brien Institute for Public Health, University of Calgary, Calgary, AB Canada; 11 Department of Community Health Sciences, University of Calgary, Calgary, AB Canada

**Keywords:** Damage control, Indications, Major trauma, Surgical procedures, operative, Systematic review

## Abstract

**Background:**

Although damage control (DC) surgery is widely assumed to reduce mortality in critically injured patients, survivors often suffer substantial morbidity, suggesting that it should only be used when indicated. The purpose of this systematic review was to determine which indications for DC have evidence that they are reliable and/or valid (and therefore in which clinical situations evidence supports use of DC or that DC improves outcomes).

**Methods:**

We searched 11 databases (1950–April 1, 2019) for studies that enrolled exclusively civilian trauma patients and reported data on the reliability (consistency of surgical decisions in a given clinical scenario) or content (surgeons would perform DC in that clinical scenario or the indication predicted use of DC in practice), construct (were associated with poor outcomes), or criterion (were associated with improved outcomes when DC was conducted instead of definitive surgery) validity for suggested indications for DC surgery or DC interventions.

**Results:**

Among 34,979 citations identified, we included 36 cohort studies and three cross-sectional surveys in the systematic review. Of the 59 unique indications for DC identified, 10 had evidence of content validity [e.g., a major abdominal vascular injury or a packed red blood cell (PRBC) volume exceeding the critical administration threshold], nine had evidence of construct validity (e.g., unstable patients with combined abdominal vascular and pancreas gunshot injuries or an iliac vessel injury and intraoperative acidosis), and six had evidence of criterion validity (e.g., penetrating trauma patients requiring > 10 U PRBCs with an abdominal vascular and multiple abdominal visceral injuries or intraoperative hypothermia, acidosis, or coagulopathy). No studies evaluated the reliability of indications.

**Conclusions:**

Few indications for DC surgery or DC interventions have evidence supporting that they are reliable and/or valid. DC should be used with respect for the uncertainty regarding its effectiveness, and only in circumstances where definitive surgery cannot be entertained.

**Supplementary Information:**

The online version contains supplementary material available at 10.1186/s13017-021-00352-5.

## Background

In patients requiring operative intervention after major trauma, surgeons must decide whether to perform a definitive or damage control (DC) procedure [1, 2]. As opposed to definitive surgery (where all injuries requiring repair are repaired and the explored cavity closed), DC surgery involves quickly controlling exsanguinating hemorrhage and/or gross contamination using one or more abbreviated (or DC) interventions [2]. The patient is subsequently admitted to the intensive care unit (ICU) for ongoing resuscitation with the goal of restoring pre-injury physiology before returning to the operating room for additional surgery [2–4].

Although widely assumed to reduce mortality in critically injured patients [5], survivors of DC surgery have been reported to have a high risk of complications (e.g., intra-abdominal sepsis, enteric fistulae, and complex ventral herniae) and often suffer long lengths of ICU and hospital stay [2, 4, 6–11]. It is therefore important to ensure that DC surgery is only performed on patients in which the expected survival benefit of the procedure outweighs its expected risk of negative consequences [1]. Despite this, the benefit/risk profile of using DC surgery in different clinical situations has not been comprehensively evaluated, and several authors have recently reported data suggesting that substantial variation in use of DC surgery exists across trauma centers or that it may be overused [12–15].

We hypothesize that variation in use of DC surgery may be at least partially explained by the uncertainty that exists as to when the procedure is indicated [1, 2]. Thus, we recently initiated a program of research to develop evidence-informed indications for the appropriate use of DC surgery and DC interventions in civilian trauma patients [1–3, 16–18]. The purpose of this systematic review and meta-analysis was to determine which indications for DC surgery and DC interventions in civilian trauma patients have evidence supporting that they are reliable and/or valid (and in which clinical situations evidence supports use of DC or that DC improves outcomes). The data reported in this study therefore provide a comprehensive assessment of the reported studies evaluating whether use of DC instead of definitive surgery is associated with improved outcomes in injured patients.

## Methods

### Protocol

Study methods were pre-specified in a protocol developed according to the Preferred Reporting Items in Systematic Reviews and Meta-analyses [19] and Meta-analysis of Observational Studies in Epidemiology [20] statements.

### Search strategy

Using published search strategies designed for identifying indications for DC surgery and DC interventions in trauma patients, we searched MEDLINE, EMBASE, PubMed, Scopus, Web of Science, and the Cochrane Library from their inception to April 1, 2019 without restrictions (see Supplementary Table 1 in our published protocol paper [1] for details of our electronic bibliographic database search strategies). We also used the PubMed “related articles” feature and searched references from included and relevant review articles and abstracts from conferences held between 2009 and 2015, including meetings of the American Association for the Surgery of Trauma (AAST), Australasian Trauma Society, Eastern Association for the Surgery of Trauma (EAST), Trauma Association of Canada, and Western Trauma Association (WTA). To identify unpublished studies, we searched 12 trauma organizational websites [1] and Google Scholar (the first 10 web pages) using combinations of the terms *trauma*, *injury*, *abbreviated surgery*, *bailout surgery*, *damage control*, *damage control surgery*, *indication*, and *predictor*.

### Study selection

Two investigators (D.J.R., N.B.) independently screened the titles and abstracts of citations identified by the search and selected articles for full-text review. We included full-text studies that reported original data on the reliability or validity of suggested indications for DC surgery or DC interventions in civilian trauma patients. We excluded studies that included only patients injured in combat or by thermal mechanisms or focused exclusively on DC for emergency general or vascular surgery or orthopedic or neurologic injuries. Study eligibility disagreements were resolved by consensus between the two investigators.

### Definitions

An indication was defined as a clinical finding/circumstance or scenario that reportedly advised use of DC surgery (or a DC intervention) over definitive surgery (or a definitive surgical intervention) [1]. DC surgery was broadly defined as a multi-step operative intervention, which included an abbreviated initial surgical procedure that aimed to rapidly control bleeding and/or gross contamination [1]. We did not predefine DC interventions. Instead, we included articles that satisfied the above criteria where an indication was reported for a surgical intervention suggested by authors to constitute DC or an abbreviated surgical technique [e.g., temporary abdominal closure (TAC)/open abdominal management after trauma laparotomy] [1, 2].

Indication reliability was defined as the degree to which the same decision to conduct DC was made when surgeons were provided the same clinical finding/scenario (test-retest) or when encountered by the same surgeon (intra-rater) or different surgeons (inter-rater). Validity included content, construct, and criterion validity (see Table 1 for detailed definitions of these measures) [21, 22]. Content validity was defined as the extent to which surgeons reported that they would perform DC in a given clinical scenario or that an indication predicted use of DC in practice [21, 22]. Construct validity referred to how well one indication or a combination of indications and demographic variables predicted poor outcomes in patients not treated with DC (i.e., the extent to which an indication was associated with a higher probability of poor outcomes in patients treated with definitive surgery and therefore should be considered as a potential indication for DC) [21, 22]. Criterion validity referred to the extent to which the utilization or conduct of DC instead of definitive surgery for one or more indications was associated with improved patient outcomes [21, 22].

**Table 1:** Definitions of indications for use of damage control content, construct, and criterion validity

Type of measurement validity	Epidemiologic definition [21 22]	Operationalized definition	Theoretical example of study evaluating indication content, construct, or criterion validity
Content	Extent to which the indication incorporates the domain of the phenomenon under study (e.g., the extent to which the indication includes clinical situations that surgeons feel may influence use of DC or that is associated with the choice to perform DC over definitive surgery)	Extent to which surgeons reported that they would perform DC in a given clinical scenario or that an indication predicted use of DC in practice	In a cross-sectional survey of surgeons, X% reported that they would perform DC when a major abdominal vascular injury was identified at laparotomy In a cohort study, the intraoperative identification of a major abdominal vascular injury was associated with OR of X (95% CI, X-X) for performing DC instead of definitive surgery in practice
Construct	Extent to which the indication corresponds to theoretical concepts (constructs) under study (e.g., if an indication has construct validity, it should be associated with poor patient outcomes when patients undergo definitive instead of DC surgery)	How well one indication or a combination of indications and demographic variables predicted poor outcomes in patients not treated with DC (i.e., the extent to which an indication was associated with a higher probability of poor outcomes in patients treated with definitive surgery and therefore should be considered as a potential indication for DC)	In a cohort study, the intraoperative identification of a major abdominal vascular injury was associated with an increased risk of mortality in patients who underwent definitive laparotomy for trauma
Criterion	Extent to which the indication related to a reference standard (e.g., the extent to which conducting DC instead of definitive surgery in that clinical situation was associated with an improvement in outcomes)	Extent to which the utilization or conduct of DC instead of definitive surgery for one or more indications was associated with improved patient outcomes	In a cohort study, use of DC instead of definitive surgery for patients with a major abdominal vascular injury was associated with an improvement in in-hospital adjusted mortality

Where CI indicates confidence interval; DC, damage control; and OR, odds ratio

### Data extraction

Two investigators (D.J.R., N.B.) independently extracted data from included studies into pilot-tested tables summarizing characteristics of the included studies and the content, construct, and criterion validity of suggested indications for DC surgery and DC interventions (Table 1). An interpreter assisted with data extraction for one Russian [23] and two Mandarin Chinese [24, 25] language studies. We extracted data on (1) study design, setting, and participants; (2) suggested indications for DC surgery or DC interventions as reported by study authors; and (3) measures of indication reliability and validity. For content validity, we extracted data on the percentage of surgeons that reported that they would perform DC in a given clinical scenario or odds ratios (ORs) or hazard ratios (HRs) [with surrounding 95% confidence intervals (CIs)] indicating the degree to which that indication predicted use of DC in practice. For construct validity, we extracted data on how well one indication or a combination of indications predicted outcomes in patients not treated with DC. Finally, for criterion validity, we extracted data on the extent to which the utilization or conduct of DC instead of definitive surgery for one or more indications was associated with patient outcomes. Outcomes of interest for the assessment of construct and criterion validity included survival, development of coagulopathy, reported measures of morbidity, and lengths of hospital and ICU stay. Outcomes were extracted at the longest follow-up duration. When both unadjusted and adjusted outcome estimates were reported, the most adjusted estimate was extracted.

### Risk of bias assessment

The same two investigators independently evaluated study risk of bias. Cohort studies were assessed using an expanded version of the Quality in Prognosis Studies tool [26, 27], which included questions regarding study participation and attrition; indication or outcome description and measurement; confounding measurement and account; whether the operative profile chosen (i.e., DC versus definitive surgery) may have varied in relation to the indication of interest; and methods and reporting of statistical analyses (see Supplemental Digital Content 1 for the operationalized list of quality domains evaluated) [26–28]. For cross-sectional studies, we evaluated sampling methods, response rates, and whether the reported methods would permit replication; sample was representative of the population; questionnaire was adequately described, pretested, and had evidence of reliability and/or validity; statistical methods; and if all respondents were accounted for [29]. The assessment of statistical analyses incorporated recommendations for appraising logistic regression models [30, 31]. Disagreements regarding risk of bias assessments were resolved by consensus.

### Data synthesis

We used directed qualitative content analysis to group unique indications into the subcategories and categories of a previously developed framework for conceptualizing indications for DC [3, 32]. We then used a vote counting scale [33] to incorporate our risk of bias assessments into the synthesis of evidence regarding whether indications were reliable and/or valid [26]. The aggregate scale summarized strength of evidence as (1) not reported, (2) inconclusive (no evidence or a low to moderate association in the setting of an overall high amount of bias in at least one quality domain), (3) a consistently strong association in the setting of an overall high amount of bias in only one quality domain, (4) a consistently low to moderate association in the setting of an overall moderate amount of bias in one or more quality domains, (5) a consistently low to moderate association with a low amount of bias in all quality domains or a consistently strong association with an overall moderate amount of bias in one or more study quality domains, and (6) a consistently strong association with a low overall amount of bias in all study domains.

### Statistical analyses

Inter-investigator agreement regarding full-text article inclusion was quantified using kappa (κ) statistics [34]. We summarized dichotomous data using counts (percentages) and compared them using ORs with 95% CIs or Fisher’s exact tests. We combined adjusted ORs for indications with similar definitions using Mantel-Haenszel-weighted DerSimonian and Laird random-effects models [35]. Heterogeneity in these estimates were assessed using *I*^2^ statistics and tests of homogeneity [36, 37]. We considered two-sided *p* values < 0.05 statistically significant. Stata MP version 13.1 (Stata Corp., College Station, TX) was used for statistical analyses.

## Results

### Study selection

**Fig. 1:**
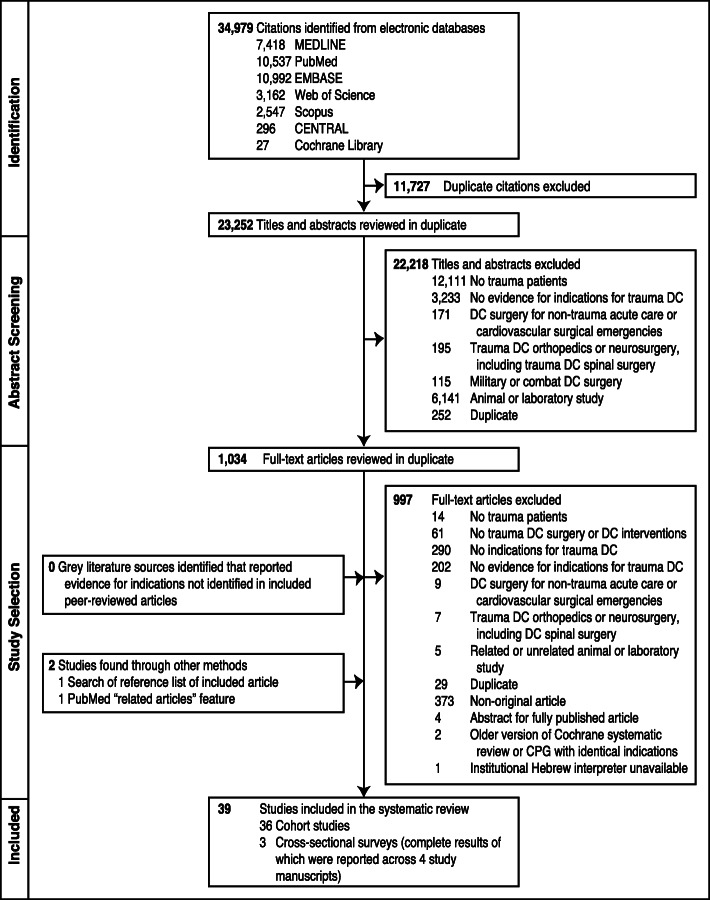
Flow of articles through the systematic review. Where CPG indicates clinical practice guideline and DC, damage control

Among 34,979 citations identified by the search, we included 36 cohort studies (*n* = 8160 total trauma patients) [14, 15, 23, 25, 38–68] and three cross-sectional surveys (*n* = 481 total surgeon respondents) [69–72] in the systematic review (Fig. 1). Agreement between investigators on full-text article inclusion was excellent (κ-statistic, 0.72; 95% CI, 0.60 to 0.83).

### Description of included studies

Characteristics of included cohort and cross-sectional studies are presented in Table 2 and Supplemental Digital Content 2, respectively. In total, 67% of the cohort studies were published on or after the year 2000 and 91.7% were conducted in single centers and 66.7% in the USA. Five (13.9%) cohort studies collected data prospectively. Studies describing age and Injury Severity Scale (ISS) scores reported enrolling severely injured (mean/median ISS score range, 17.8–41) young adults (mean/median age range, 26–51.9 years).

**Table 2:** Characteristics of the 36 cohort studies included in the systematic review

Source	Prospective	No. centers	Country	Trauma Patients					No. indications
				No.	Mean age, yr.	% blunt MOI	Mean ISS Score	Predominant trauma surgical intervention(s) performed^a^ (%)	
Watson et al. 2017 [15]	Yes	12	U.S.A.	329	32.0^b^	42	26.0^b^	DC laparotomy (65) or definitive surgery (35)	2
Harvin et al. 2016 [38]	No	1	U.S.A.	222	33.6	66	28.7	DC (65) or definitive (35) laparotomy	6
Savage et al. 2014 [39]	No	1	U.S.A.	169	40.2	65	NISS, 21.1	DC (22) or definitive (78) laparotomy	2
Ordoñez et al. 2014 [40]	No	1	Colombia	36	26^b^	0	25^b^	DC (81) or definitive (19) laparotomy for complex penetrating duodenal injuries	1
Mahmood et al. 2014 [41]	Yes	1	Qatar	117	35	92	23	DC (13) or definitive (87) laparotomy	5
Thompson et al. 2013 [42]	No	1	U.S.A.	15	29^b^	33	35^d^	DC laparotomy with or without the first stage of a Whipple procedure (80) versus a complete Whipple procedure at the index operation (20)	1
Rice et al. 2012 [43]	Yes	100	26 countries	556	38.1	85	NR	DC or definitive surgery (percentages unclear)	3
Martin et al. 2012 [14]	No	1	U.S.A.	628	34.5	45	17.8	DC (10) or definitive (90) laparotomy	3
Chinnery et al. 2012 [44]	No	1	South Africa	219	26^b^	0	NR	DC (20) or definitive (80) laparotomy	1
Mayberry et al. 2011 [45]	No	1	U.S.A.	41	34	46	24	DC (61) or definitive (39) laparotomy for full-thickness duodenal lacerations	1
Liu et al. 2011 [24]	No	1	China	104	31.1	84	NR	DC (44) or definitive (56) laparotomy	4
Leppäniemi et al. 2011 [46]	No	2	Finland	144	33	94	31	DC (15) or definitive (20) laparotomy for major liver injury	1
Timmermans et al. 2010 [47]	No	1	South Africa	74	28	19	NR	DC laparotomy (100)	3
Matsumoto et al. 2010 [48]	No	1	Japan	34	51.9	97	35.6	DC laparotomy (100)^c^	6
Kairinos et al. 2010 [49]	No	1	South Africa	145	NR	NR	NR	DC laparotomy (100)	1
Yu et al. 2009 [25]	No	1	China	90	NR	NR	35	DC (50) or definitive (50) surgery	7
Kashuk et al. 2008 [50]	No	1	U.S.A.	133	34.9	NR	35.9	NR	4
MacKenzie et al. 2004 [51]	No	1	Canada	37	NR	84	NR	Laparotomy with early therapeutic perihepatic packing followed by angioembolization (19) or definitive laparotomy (81) for AAST grade IV-V liver injuries^d^	1
Asensio et al. 2004 [52]	Mixed	1	U.S.A.	139	33.2	28	24.0	DC laparotomy (100)	6
Aucar et al. 2003 [53]	Yes	1	U.S.A.	31	NR	NR	NR	DC (23) or definitive (77) operative techniques for patients with chest, abdomen, or extremity vessel injuries	3
Asensio et al. 2003 [54]	No	1	U.S.A.	148	27.0	5	20.0	DC (18) or definitive (82) laparotomy for iliac vessel injuries	4
Hirshberg et al. 2002 [55]	Mixed	1	U.S.A.	346	NR	0	NR	DC (16) or definitive (84) laparotomy	2
Apartsin et al. 2002 [23]	No	1	Russia	150	NR	NR	NR	DC (51) or definitive (49) laparotomy	2
Asensio et al. 2001 [56]	No	1	U.S.A.	548	30	18	32	DC laparotomy (100)	6
Krishna et al. 1998 [57]	No	1	New Zealand	40	38.2	NR	>35	Definitive thoracotomy (10) and/or laparotomy (93)	3
Carrillo et al. 1998 [58]	No	1	U.S.A.	64	36	0	NR	DC (22) or definitive (78) laparotomy for iliac vessel injuries	6
Cushman et al. 1997 [59]	No	1	U.S.A.	53	29	5	NR	DC (17) or definitive (83) laparotomy for iliac vessel injuries	6
Cosgriff et al. 1997 [60]	Yes	1	U.S.A.	58	35.4	47	30.6	DC (31) or definitive (69) laparotomy	4
Garrison et al. 1996 [61]	No	1	U.S.A.	70	41	70	35.3	DC laparotomy with therapeutic intra-abdominal packing (100)	7
Rotondo et al. 1993 [62]	No	1	U.S.A.	46	31.0	0	23.6	DC (52) or definitive (48) laparotomy	1
Sharp and Locicero 1992 [63]	No	1	U.S.A.	39	33.9	80	37.9	DC laparotomy with therapeutic intra-abdominal packing (100)	6
Rutherford et al. 1992 [64]	No	1	U.S.A.	3038	NR	85	NR	NR	3
Burch et al. 1992 [65]	No	1	U.S.A.	200	31	15	NR	DC laparotomy (100)	2
Cué et al. 1990 [66]	No	1	U.S.A.	35	35	71	41	Laparotomy (100) with packing of major liver injuries (89) or severe retroperitoneal hemorrhage (11)	1
Carmona et al. 1984 [67]	No	1	U.S.A.	31	30^e^	53^e^	NR	Laparotomy with perihepatic packing (55) or simple repair (45) of major liver injuries (55)	1
Stone et al. 1983 [68]	No	1	U.S.A.	31	28	6	NR	DC (55) or definitive (45) laparotomy	1

Where AAST indicates American Association for the Surgery of Trauma; d, day(s); DC, damage control; ED, Emergency Department; ICU, Intensive Care Unit; intraop, intraoperative; ISS, Injury Severity Scale; MSK, musculoskeletal/extremity; NA, not applicable; NISS, New Injury Severity Scale score; NR, not reported; OR, operating room; preop, preoperative; pro, prospective; ret, retrospective; RR, response rate; TAC, temporary abdominal closure; and yr, years.

^a^Excluding orthopedic and neurological surgery

^b^Value represents a median instead of a mean

^c^In this study, 16 (47%) of the 34 patients who underwent DC laparotomy had their procedure performed in the ED

^d^In this study 15 of the patients in the definitive laparotomy group were reported to ultimately need abdominal packing after conventional hepatic injury repair techniques. Moreover, 1 patient in the early therapeutic packing group received angiography before laparotomy

^e^Estimate relates to the 17 patients who were managed with therapeutic perihepatic packing

The three cross-sectional studies described results of two surveys of AAST members (administered in 1997–1998 [72] and 2005 [69]) and one of Trauma Association of Canada surgeon members (administered in 2004; results of which were reported across two publications) [70, 71]. Surgeon response rates varied from 26% [69] to 84% [70, 71]. Of the respondents, two studies reported that 73–85% practiced in academic centers [69, 72] and one that 45% specialized in trauma and critical care [69].

### Risk of bias assessment

The risk of bias assessment for the 36 included cohort studies is outlined in Supplemental Digital File 3. Nine (25.0%) studies were at high risk of study participation bias, 17 (47.2%) reported outcome comparisons at high risk of confounding, and 28 (77.8%) did not report follow-up duration. Six of the nine studies at high risk of study participation bias evaluated the construct validity of indications among patients treated only with DC surgery (and therefore outcomes associated with these variables are better interpreted as predictors of poor outcome after DC surgery rather than measures of indication construct validity) [47, 48, 56, 61, 63, 66]. Of the 17 cohort studies that adjusted estimates using logistic regression, these analyses were at a moderate risk of bias in 12 studies and a high risk of bias in one study, largely because of a low or unclear number of events per variable and inadequate reporting of methods used to select predictor variables and/or build models (Supplemental Digital Content 4).

The risk of bias assessment for the three included cross-sectional studies is included in Supplemental Digital File 5. Two studies described methods that would permit replication, identified a sample that was likely representative of the broader trauma surgical community, and adequately described the questionnaire (or provided it as supplementary material) [70–72]; however, only one reported that the questionnaire was pretested [70, 71] and none provided evidence of questionnaire reliability or validity.

### Reliability and validity of indications for use of DC surgery and DC interventions

The 39 included studies assessed the content, construct, and criterion validity of 116 indications for DC surgery (median per article, 3; range, 1–7) and 32 indications for TAC/open abdominal management (median per article, 7; range, 1–12). No studies evaluated the reliability of indications for DC surgery or DC interventions.

#### Content validity

The cross-sectional studies [69–72] evaluated the content validity of indications for TAC after trauma laparotomy by asking surgeons whether open abdominal management was indicated in different clinical situations (see Table 3 for assessments of content validity in cross-sectional and cohort studies). Most respondents supported use of TAC when they were unable to close the fascia (or closure was subjectively tight), there was massive visceral edema, planned reoperation was required (e.g., to remove packs or perform a “re-look” laparotomy), or signs of abdominal compartment syndrome developed upon attempted fascial closure [69–72].

**Table 3:** Content validity (surgeons would performed damage control in that clinical scenario or the indication predicts use of damage control in practice) of reported indications for use of damage control surgery or damage control interventions in civilian trauma patients

Indication (variable included in statistical analyses)	Confounding factors adjusted for	Outcome	Content validity
Surgeon opinions on the content validity of indications for use of TAC/open abdominal management after laparotomy in cross-sectional studies			
Prep for a second look [69]	NA	Percentage of respondents who would leave the abdomen open	6%
Abdominal organ distention [69]			22%
Inability to close the fascia [69]			20%
Physically unable to close the fascia [70]	NA	Percentage of respondents supporting relevance of indications for leaving the abdomen open after trauma laparotomy	87%
Planned reoperation [70]			80%
Intra-abdominal packing [70]			59%
Magnitude of injury/gestalt [70]			43%
Airway pressure measurements [70]			41%
Bladder pressure measurements [70]			39%
Visual edema of the bowel [70]			33%
Young previously healthy male; grade IV spleen injury identified at laparotomy; massive hemoperitoneum (20% blood volume loss); no other intra- or extra-abdominal injuries; 45 min laparotomy; given 4 L crystalloid and 4 U PRBCs; intraoperative temperature 36.2 °C, pH 7.34; INR 1.3; and [71]	NA	Percentage of respondents who would perform TAC^a^	
Fascial closure possible without excessive tension			1%
Fascial closure extremely tight			45%
Fascial closure physically not possible but skin closure is			51%
Neither fascial nor skin closure is possible			73%
Same as the above scenario except a splenectomy was performed; intraoperative temperature 34 °C, pH 7.16, and INR 2.0; and [71]			
Fascial closure possible without excessive tension			9%
Fascial closure extremely tight			61%
Fascial closure physically not possible but skin closure is			50%
Neither fascial nor skin closure is possible			75%
Young previously healthy male; presented with severe hemorrhagic shock (40% blood volume loss); bleeding grade III stellate liver rupture with devitalization of 30% of the right hepatic lobe, grade IV spleen injury which is no longer bleeding, 6 cm diaphragmatic tear, devascularization of a 6 cm segment of small bowel, and a one-third thickness circumferential tear of the distal descending colon; after packing of liver and spleen and repair of the diaphragm, major bleeding appears controlled, but there is diffuse oozing from cut surfaces; BP is 80/40 mmHg with vasopressors and after infusion of 8 L of crystalloid and 16 U PRBCs; intraoperative temperature 34 °C, pH 7.16, and INR 2.0; and fascial closure without tension is possible [71]			75%
Subjectively tight closure [72]	NA	Percentage of respondents who were much less or less willing to close the abdomen after trauma laparotomy	77%
Massive bowel edema [72]			89%
Multiple intra-abdominal injuries [72]			21%
Intra-abdominal packing [72]			71%
Fecal contamination/peritonitis [72]			12%
Massive transfusion [72]			19%
Hypothermia [72]			21%
Acidosis (pH < 7.3) [72]			22%
Coagulopathy [72]			31%
Planned reoperation [72]			76%
Pulmonary deterioration on closure [72]			94%
Hemodynamic instability with closure [72]			91%
Association between indications and use of DC in practice [as predicted by cohort studies estimating the association (e.g., OR or HR for conducting DC) between certain clinical scenarios and the decision to conduct DC in practice]			
*Preoperative indications*			
High ISS [15]	Study site, penetrating mechanism, major abdominal vascular injury	Use of DCL	OR per ISS ↑, 1.05 (95% CI, 1.02–1.07)
Systolic BP < 90 mmHg on admission and grade III–V liver injury [46]	None	Use of DCL	Not associated with use of DCL
An artificial neural network including variables for bullet wound location (right or left chest or upper or lower abdominal quadrant) and trajectory pattern [horizontal shift (e.g., one that traversed the abdomen from RUQ to LUQ) or entry wound in back] and lowest ED systolic BP predicted that DC laparotomy would be used in patients with a horizontal shift upper abdominal trajectory pattern and a systolic BP < 105 mmHg or a RUQ wound with a bullet retained in the same quadrant and a systolic BP < 90 mmHg [55]	Bullet wound location and trajectory pattern, lowest ED systolic BP	Use of DCL	Model Se, 83%; model Sp, 93%
*Intraoperative indications*			
Major abdominal vascular injury [15]	Study site, ISS, penetrating mechanism	Use of DCL	OR, 2.70 (95% CI, 1.42-5.16)
Combined AAST grade III–V liver and IV–V spleen injury [46]	None	Use of DCL	All patients with this injury pattern underwent DCL while 42% of those without it did not (*p* = 0.02)
AAST grade V liver injury [46]	NR	Use of DCL	Not associated with ↑ use of DCL when compared to patients with grade III-IV injury
*Pre*- *or intraoperative indications* (*or indications for which the setting was unclear or not specified*)			
Multiple trauma and AAST grade III-V liver injury [46]	NR	Use of DCL	Not associated with use of DCL
Transfusion > 10 U PRBCs and AAST grade III-V liver injury [46]	NR	Use of DCL	Not associated with use of DCL
Transfusion of a large volume of PRBCs [41]	FFP and fluids administered, BD, lactate	Use of DCL	OR per PRBC U ↑, 1.05 (95% CI, 0.85–1.29)
Transfusion of a large volume of FFP [41]	PRBCs and fluids administered, BD, lactate	Use of DCL	OR per FFP U ↑, 0.95 (95% CI, 0.77–1.18)
Administration of a large volume of fluids [41]	PRBCs and FFP administered, BD, lactate	Use of DCL	OR per L of fluids ↑, 1.13 (95% CI, 0.92–1.37)
A PRBC transfusion volume that exceeds the CAT [39]	Admission systolic BP, MOI, NISS	Use of DCL	HR, 2.72 (95% CI, 1.26–5.91)
The number of times the PRBC transfusion volume exceeds the CAT [39]	Admission systolic BP, MOI, NISS	Use of DCL	HR per CAT multiple, 1.27 (95% CI, 1.11–1.47) (survival was 89.3%, 66.7%, 64.3%, and 75% in CAT0, CAT1, CAT2, and CAT3 pts, respectively )[39]
Elevated BD (max BD) [41]	PRBCs, FFP, and fluids transfused, lactate	Use of DCL	OR per max BD ↑, 1.25 (95% CI, 0.97–1.61)
Elevated lactate (max lactate) [41]	PRBCs, FFP, and fluids transfused, BD	Use of DCL	OR per max lactate ↑, 0.94 (95% CI, 0.73–1.22)

Where AAST indicates American Association for the Surgery of Trauma; BD, base deficit; BP, blood pressure; CAT, critical administration threshold (≥ 3 units of packed red blood cells administered in 1 h of the first 24 h of injury); DC, damage control; DCL, damage control laparotomy; ED, Emergency Department; GSW, gunshot wound; ISS, Injury Severity Scale score; INR, international normalized ratio; LUQ, left upper quadrant; pts, patients; NA, not applicable; PPV, positive predictive value; PRBCs, packed red blood cells; RUQ, right upper quadrant; Se, sensitivity; Sp, specificity; and TAC, temporary abdominal closure

^a^The definition of TAC in this study did not include mesh fascial closures

Of the cohort studies that evaluated indication content validity (i.e., whether the indication predicted use of DC in practice), Hirshberg et al. reported that an artificial neural network containing torso bullet wound location/trajectory pattern and systolic blood pressure (BP) in the emergency department (ED) had a high sensitivity (83%) and specificity (93%) for identifying patients treated with DC laparotomy [55]. Further, Watson et al. reported that a major abdominal vascular injury was independently associated with the decision to conduct DC laparotomy among patients enrolled in in the Pragmatic, Randomized Optimal Platelet and Plasma Ratios (PROPPR) randomized trial [15]. Another study by Leppäniemi et al. reported that all patients with a AAST grade III–V liver and IV–V spleen injury underwent DC laparotomy while 42% of those without this injury pattern did not (*p* = 0.02) [46]. Finally, Savage et al. reported that packed red blood cell (PRBC) transfusion volumes exceeding the critical administration threshold (≥ 3 U PRBCs in 1 hour of the first 24 h of injury) were independently associated with a dose-dependent increase in use of DC laparotomy [39].

#### Construct validity

The construct validity of indications (or predictive models containing a combination of indications, other clinical findings, and/or baseline demographic variables) for DC surgery was evaluated in 23 studies, which examined associations between indications and survival or coagulopathy (Table 4). Three studies reported that a high ISS score, preoperative hypothermia, an elevated base deficit in the pre- or intraoperative setting, and the identification of a combined pancreas and abdominal vascular injury during operation were independently associated with decreased survival in patients mostly treated with definitive surgery, suggesting that DC should be considered in these high-risk scenarios [44, 57, 64]. Interestingly, however, a preoperative pH < 7.20 was also independently associated with decreased survival among injured patients who received DC laparotomy in another study (suggesting that it was also a poor prognostic factor among those treated with DC) [47]. Two other studies evaluated the association between development of a laboratory-confirmed coagulopathy (variably defined), a clinical scenario where DC has long been recommended over definitive surgery, and an ISS score > 25, systolic BP < 70 mmHg, or lowest temperature < 34 °C or pH < 7.1 in trauma patients transfused > 10 units of PRBCs in the first 6 or 24 h [50, 60]. Among these studies, the pooled adjusted OR for development of a laboratory-confirmed coagulopathy among patients with an ISS score > 25 was 6.11 (95% CI, 1.68–22.16; *I*^2^ = 0%; heterogeneity *p* = 0.65), systolic BP < 70 mmHg was 1.66 (95% CI, 0.15–19.10; *I*^2^ = 79.5%; heterogeneity *p* = 0.03), lowest temperature < 34 °C was 7.12 (95% CI, 2.53–20.05; *I*^2^ = 0%; heterogeneity *p* = 0.74), and lowest pH < 7.1 was 4.14 (95% CI, 0.60–28.67; *I*^2^ = 74.2%; heterogeneity *p* = 0.05).

**Table 4:** Construct validity (how well one indication or a combination of indications and demographic variables predicted patient outcomes in patients not treated with damage control) of reported indications for use of damage control surgery in civilian trauma patients

Indication (variable included in statistical analyses)	% DC pts in the study	Confounding factors adjusted for	Outcome	Predictive validity
Preoperative indications				
*Overall injury burden*				
High ISS	0	BD, temperature	Survival	BD, temperature OR per ISS ↑, 1.12 (95% CI, 1.03 to 1.23) [57]
	100	None	Survival	↑ mean ISS in non-survivors vs. survivors (38 vs. 29, *p* < 0.05) [61]
*Volume and*/*or type of resuscitation provided*				
Transfusion of a large volume of PRBCs	100	None	Survival	↑ mean U PRBCs transfused in non-survivors vs. survivors (20 vs. 14, *p* < 0.01) [61]
*Degree of physiologic insult*				
Prolonged duration of hypotension	100	None	Survival	↑ mean duration of preoperative hypotension in non-survivors vs. survivors (90 vs. 50 min., *p* < 0.05) [61]
Hypothermia (min temperature)	0	BD, ISS	Survival	OR per min temperature ↓ in °C, 0.32 (95% CI, 0.15 to 0.64) [57]
Temperature < 35 °C	100	Age, BD, pH	Survival	↓ temperature not independently associated with survival [47]
Elevated BD (max BD)	0	ISS, temperature	Survival	OR per max BD ↑, 0.66 (95% CI, 0.56 to 0.78) [57]
BD > 10.5 mEq/L	100	Age, pH, temperature	Survival	↑ BD not independently associated with survival [47]
Decreased pH	100	None	Survival	↓ mean pH in non-survivors vs. survivors (7.1 vs. 7.3, *p* < 0.05) [61]
pH < 7.20	100	Age, BD, temperature	Survival	↑ pH independently associated with ↓ survival (*p* = 0.001) [47]
Decreased platelet count	100	None	Survival	↓ mean platelet count in non-survivors vs. survivors (179,000 vs. 229,000 mm^3^) [61]
Laboratory-confirmed coagulopathy	100	None	Survival	↑ mean PT (22 vs. 14 s) and PTT (69 vs. 42 s) in non-survivors vs. survivors (*p* < 0.05 for both) [61]
PT ≥ 16 s	100	None	Survival	OR, 0.11 (*p* < 0.05) [63]
PTT ≥ 50 s	100	None	Survival	OR, 0 (survival, 0% vs. 71% with PTT <50 sec; *p* < 0.05) [63]
A model included highest ED BD, lowest ED temperature, and ISS	0	BD, lowest ED temperature, ISS	Survival	Model Se, 83%; model Sp, 93% [57]
A model predicting that survival was possible only when the equation 0.012(age) - 0.707(lowest preoperative pH) - 0.032(lowest preoperative temperature in °C) + 6.002 = < 0.5	100	None	Survival	Model Se, 25%; model PPV, 100% [49]
Intraoperative indications				
*Injury pattern identified during operation*				
Combined abdominal vascular and pancreas gunshot injuries	20	12 variables^a^	Survival	OR, 0.12 (95% CI, 0.041–0.36) [44]
	20	11 variables^a^	Complications	OR, 3.59 (95% CI, 1.10–11.68) [44]
Iliac vessel injury and prolonged duration of hypotension	22	None	Survival	↑ mean duration of hypotension in non-survivors vs. survivors who underwent definitive (95 vs. 65 min, *p* value NR) and DC (40 vs. 85 min, *p* < 0.05) laparotomy [58]
Iliac vessel injury and *initial* temperature < 34 °C	17	None	Survival	OR, 0.27 (95% CI, 0.072-1.0) [59]
Iliac vessel injury and *final* temperature < 35 °C	17	None	Survival	OR, 0.025 (95% CI, 0.0028-0.23) [59]
Iliac vessel injury and *initial* BD > 15 mEq/L	17	None	Survival	OR, 0.037 (95% CI, 0.0072-0.19) [59]
Iliac vessel injury and *final* BD > 6 mEq/L	17	None	Survival	OR, 0.091 (95% CI, 0.019–0.45) [59]
Iliac vessel injury and *initial* pH < 7.1	17	None	Survival	OR, 0.032 (95% CI, 0.0055–0.19) [59]
Iliac vessel injury and *final* pH < 7.3	17	None	Survival	OR, 0.069 (95% CI, 0.014–0.36) [59]
Penetrating iliac vessel injury and *final* pH < 7.2	22	None	Survival	↓ mean final pH in non-survivors vs. survivors who underwent definitive (7.11 vs. 7.29, *p* value NR) and DC (7.20 vs. 7.32, *p* value < 0.05) [58]
Penetrating iliac vessel injury and *final* PT > 20 s	22	None	Survival	↑ final PT in non-survivors vs. survivors who underwent definitive (25.2 vs. 17.8 sec, *p* value NR) and DC (20.2 vs. 15.9 s, *p* < 0.05) laparotomy [58]
Penetrating iliac vessel injury and *final* PTT >70 s	22	None	Survival	↑ final PTT in non-survivors vs. survivors who underwent definitive (86.1 vs. 59.2 s, *p* value NR) and DC (66.2 vs. 47.8 s, *p* < 0.05) laparotomy [58]
Iliac vessel injury and shock, hypothermia, acidosis, or coagulopathy (timing of measurement not specified)	18	≤ 14 variables^b^	Survival	Shock, hypothermia, acidosis, and coagulopathy not independently associated with survival [54]
*Volume and*/*or type of resuscitation provided*				
Transfusion > 4 L PRBCs	100	23 variables^b^	Survival	Independently associated with ↓ survival [56]
Transfusion > 5 L PRBCs and whole blood	100	None	Survival	↑ mean volume of PRBCs and whole blood in non-survivors vs. survivors (8.2 vs. 5.6 L, *p* < 0.001) [56]
Administration > 12 L PRBCs and/or whole blood, other blood products, and crystalloids	100	None	Survival	↑ mean volume of these fluids in non-survivors vs. survivors (15.0 vs. 12.4 L, *p* < 0.001) [56]
*Degree of physiologic insult*				
Temperature ≤ 34 °C	100	None	Survival	↓ mean min temperature in non-survivors vs. survivors (33.9 vs. 35.0, *p* < 0.001) [56]
Serum [HCO_3_^-^] ≤ 15 mEq/L	100	None	Survival	Serum [HCO_3_^-^] ≤ 15 mEq/L associated with ↓ survival [56]
pH < 7.2	100	None	Survival	↓ mean initial (7.1 vs. 7.4), max (7.2 vs. 7.4), and min (7.0 vs. 7.2) pH in non-survivors vs. survivors (*p* < 0.001 for all) [56]
Elevated ACT	23	Unclear for logistic regression	Clinical coagulo-pathy^c^	The mean of 2 ACT measurements (taken within the first 10 min of beginning surgery and repeated ~ 15 min later) was 180 s in patients with coagulopathy versus 118 s in those without (*p* < 0.001) [53] The 1^st^, 2^nd^, and mean ACT values were independently associated with coagulopathy using logistic regression (*p* value NR) [53]
Systolic BP < 90 mmHg, BD > 7.5 mEq/L, and/or temperature < 35.5 °C at the start of surgery	100	None	Survival	OR for survival was 0.13 (95% CI, 0.021-0.77) among patients who presented with all 3 vs. < 3 variables. There was also a stepwise ↓ in survival as the no. of variables present ↑ [48]
A model predicted that survival was only possible when patients lie below and to the right of a diagonal discriminant line given by the equation PRBC transfusion rate (U/h) = 35.7(arterial pH) - 242 (for an arterial pH = 7.2, transfusion rate = 15 U/h)	100	PRBC transfusion rate, pH	48 h survival	Model Se, 77% [65]
Pre- or intraoperative indications (or indications for which the setting was unclear or not specified)				
*Volume and/or type of resuscitation provided*				
Transfusion > 15 U PRBCs	100	None	Coagulopathy^d^	OR, 6.0 (95% CI, 0.67–75.61) [66]
Transfusion > 10 U PRBCs in the first 24 h and an ISS > 25	31	PRBCs transfused in 24 h, lowest systolic BP < 70 mmHg, pH < 7.1, and temperature < 34 °C	PT & PTT > 2^a^control	OR, 7.7 (95% CI, 1.5–38.8) [60]^e^
Transfusion > 10 U PRBCs in the first 6 h and an ISS >25	NR	9 variables^f^	INR > 1.5 at 6 h	OR, 4.14 (95% CI, 0.57–3.18) [50]
Transfusion > 10 U PRBCs in the first 24 h and the lowest systolic BP < 70 mmHg	31	ISS > 25, PRBCs transfused in 24 h, pH < 7.1, and temperature < 34 °C	PT & PTT > 2^a^control	OR, 5.8 (95% CI, 1.2–28.2) [60]^e^
Transfusion > 10 U PRBCs in the first 6 h and ED systolic BP < 70 mmHg	NR	9 variables^f^	INR > 1.5 at 6 h	OR, 0.48 (95% CI, 0.10–2.23) [50]
Transfusion > 10 U PRBCs in the first 24 h and lowest temperature < 34 °C	31	ISS > 25, PRBCs transfused in 24 h, lowest systolic BP <70 mmHg, and pH <7.1	PT & PTT > 2^a^control	OR, 8.7 (95% CI, 1.8–41.8) [60]^e^
Transfusion > 10 U PRBCs in the first 6 h and ED temperature < 34 °C	NR	9 variables^f^	INR > 1.5 at 6 h	OR, 6.10 (95% CI, 1.54–24.19) [50]
Transfusion > 10 U PRBCs in the first 24 h and lowest pH < 7.1	31	ISS > 25, PRBCs transfused in 24 h, lowest systolic BP < 70 mmHg, and temperature < 34 °C	PT & PTT > 2^a^control	OR, 12.3 (95% CI, 2.4–64.0) [60]^e^
Transfusion > 10 U PRBCs in the first 6 h and ED pH < 7.1	NR	9 variables^f^	INR > 1.5 at 6 h	OR, 1.69 (95% CI, 0.56–5.08) [50]
*Degree of physiologic insult*				
Min temperature ≤ 33 °C	100	None	Survival	OR, 0.20 (p-value reported as NS) [63]
Elevated max BD in the first 24 h in blunt trauma patients without TBI	NR	Age ≥ 55 yr	Mortality	OR per max BD ↑, 1.39 (95% CI, 1.35 to 1.41) [64]
Elevated max BD in the first 24 h in penetrating trauma patients without TBI	NR	Age ≥ 55 yr	Mortality	OR per max BD ↑, 1.58 (95% CI, 1.44 to 1.75) [64]
Elevated max BD in the first 24 h in blunt trauma patients with TBI	NR	Age ≥ 55 yr	Mortality	OR per max BD ↑, 1.25 (95% CI, 1.14 to 1.38) [64]
Min pH ≤ 7.18	100	None	Survival	OR, 0.17 (*p* < 0.05) [63]
*Miscellaneous*				
Transfusion ≥ 10 U PRBCs, lowest ED or intraoperative temperature ≤ 33 °C, pH ≤ 7.18, ED PT ≥ 16 s, or ED PTT ≥ 50 s	100	None	Survival	↓ survival when 4–5 (0% vs. 82%; *p* < 0.04) or 2–3 (17% vs. 82%; *p* < 0.003) vs. 0–1 of these indications were present [63]
A model including BD, penetrating MOI, TBI, age ≥ 55 yr, and an interaction between BD and penetrating MOI and BD and TBI. This model predicted that the BD for which the probability of survival was 75% was 15 mmol/L for young patients without TBI versus 8 mmol/L for patients aged < 55 yr with a TBI and older patients aged ≥ 55 yr	NR	BD, penetrating MOI, TBI, age ≥ 55 yr	75% survival	Model Se, 71%; model Sp, 89% [64]

Where ACT, activated coagulation time; BD, base deficit; BP, blood pressure; CI, confidence interval; DC, damage control; ED, Emergency Department; FFP, fresh frozen plasma; h, hours; HD, hemodynamic; INR, international normalized ratio; ISS, Injury Severity Scale score; max, maximum; min, minimum; MOI, mechanism of injury; NISS, New Injury Severity Scale score; NR, not reported; NS, not significant; OR, operating room; PPV, positive predictive value; PRBCs, packed red blood cells; PT, prothrombin time; PTT, partial thromboplastin time; pts, patients; U, unit(s); Se, sensitivity; Sp, specificity; ULN, upper limit of normal; and yr, years

^a^Variables reported to be entered into the logistic regression model for mortality included age; Revised Trauma Score; systolic BP < 90 mmHg on admission; need for a major transfusion and volume transfused; need for DC surgery; AAST grade III-V pancreas injury and proximal pancreas injury; associated colonic, duodenal, and vascular injuries; postoperative complications; ICU admission; and length of ICU stay. Variables reported to be entered into the logistic regression model for complications included age; Revised Trauma Score; systolic BP < 90 mmHg on admission; need for transfusion and volume of blood transfused; need for DC surgery; grade of pancreas injury; repeat laparotomy; second pancreatic surgery; associated duodenal or vascular injury; intensive care unit (ICU) admission; and length of ICU stay

^b^Variables reported to be entered into the regression model included those associated with mortality (*p* < 0.20) that did not have > 10% missing data. These may have included, at a minimum, systolic BP and respiratory rate in the ED; Glasgow Coma Scale score, ISS, and Revised Trauma Scale score; preoperative hematocrit; crystalloids and blood given in the ED; estimated intraoperative blood loss; crystalloids and blood given in the OR; total fluids; and length of stay in the surgical intensive care unit and hospital

^b^Variables reported to be entered into the logistic regression model included those associated with survival in bivariate analysis (*p* < 0.20). These appeared to at least include ISS > 20; RTS > 0; GCS ≤ 3 or < 9; MOI; absence of spontaneous ventilation, a palpable carotid pulse, or extremity movement; non-sinus rhythm on the electrocardiogram; systolic BP and respiratory rate as a 3-level or 2-level categorical variable; a pulmonary artery and vein, thorax, thoracic or abdominal vascular, or liver injury; thoracotomy or laparotomy in the OR; coagulopathy; dysrhythmia; and type of dysrhythmia

^c^Defined by the authors as the perceived need to initiate DC maneuvers by a surgical attending, which was reported to be subjective, but usually occurred in the setting of major blood loss, hypothermia, acidosis, and the presence of multiple injuries [53]

^d^Defined by the authors as diffuse bleeding from all wounds without discrete bleeding vessels, absence of observable clots, prolonged PT and PTT along with decreased platelet count, or decreased platelet count alone [66]

^e^In this study, the probability of developing coagulopathy (defined as a PT and PTT > 2 times that of normal laboratory control) in patients who had received a transfusion of > 10 Us PRBCs in the first 24 h was 10% for those with an ISS > 25; 39% for those with an ISS > 25 and lowest systolic BP < 70 mmHg; 58% for those with an ISS > 25 and lowest pH < 7.1; 49% for those with an ISS > 25 and lowest temperature < 34 °C; 85% for those with an ISS > 25 and lowest systolic BP < 70 mmHg and temperature < 34 °C; and 98% for those with an ISS > 25 and lowest systolic BP < 70 mmHg, pH < 7.1, and temperature < 34 °C.

^f^Variables entered into the logistic regression model included FFP:PRBC ratio at 6 h; age > 55 years; ISS > 25; PRBC, FFP, and platelet U transfused at 6 h; crystalloids in 24 h; and ED systolic BP < 70 mmHg, temperature < 34 °C, and pH < 7.1

#### Criterion validity

Two studies evaluated outcomes associated with implementation or utilization of indications for DC surgery while 14 compared outcomes of patients treated with DC versus definitive surgery in different clinical situations (Table 5). Rice et al. reported that, when compared to only minor deviations, moderate or major deviations from a protocol that suggested use of DC surgery in injured patients with a temperature < 35 °C, lactate > 4 mmol/L (or more than twice the upper limit of normal), or corrected pH < 7.3 was independently associated with reduced survival [43]. Asensio et al. reported that implementing a guideline that suggested use of DC surgery for trauma patients with one of 12 different clinical findings/events was associated with a decreased unadjusted odds of infections, an increased unadjusted odds of abdominal wall closure, and reduced unadjusted lengths of ICU and hospital stay [52].

**Table 5:** Criterion validity (extent to which the utilization or conduct of damage control instead of definitive surgery for one or more indications was associated with patient outcomes) of reported indications for use of damage control surgery or damage control interventions

Source	Treatment or exposure group (*n*)	Comparison group (*n*)	Confounding factors adjusted for	Outcome(s)
Harvin et al. 2016 [38]	DC laparotomy (*n* = 144) for intra-abdominal packing (68%), a second-look laparotomy (6%), hemodynamic instability (15%), to expedite postoperative care or intervention (8%), abdominal compartment syndrome prophylaxis (1%), contamination (1%), or for other/unclear reasons (1%)	Definitive laparotomy (*n* = 78)	Propensity scores created using 17 different variables^a^	The adjusted incidence of ileus was 13% (95% CI, 6–26%) higher in the DC versus definitive laparotomy group The adjusted incidence of suture line failure was 7% (95% CI, 0–14%) higher in the DC versus definitive laparotomy group The adjusted incidence of GI bleed was 4% (95% CI, 0–7%) higher in the DC versus definitive laparotomy group The adjusted incidence of abdominal fascial dehiscence was 11% (95% CI, 2–19%) higher in the DC versus definitive laparotomy group The adjusted incidence of superficial SSI was 19% (95% CI, 10–28%) higher in the DC versus definitive laparotomy group The adjusted incidence of death was 18% (95% CI, 11–26%) higher in the DC versus definitive laparotomy group
Ordoñez et al. 2014 [40]	DC laparotomy with primary duodenal repair (*n* = 14) for complex penetrating AAST grade II–IV duodenal injuries	Definitive laparotomy with primary duodenal repair for complex penetrating AAST grade II-IV duodenal injuries (*n* = 7)	None	Survival was 92.9% in the DC versus 100% in the definitive laparotomy group (*p* > 0.99)
Thompson et al. 2013 [42]	DC surgery with or without the first stage of a Whipple procedure for patients with severe pancreaticoduodenal trauma (*n* = 12)	A complete Whipple procedure (including reconstruction) at the index operation for patients with severe pancreaticoduodenal trauma (*n* = 3)	None for all comparisons	Survival was 83.3% in the DC versus 100% in the complete Whipple group (*p* > 0.99) Sepsis occurred in 16.7% of the DC versus 100% of the complete Whipple group (*p* = 0.01) Enterocutaneous/enteroatmospheric fistulae occurred in 8.3% of the DC versus 66.7% of the complete Whipple group (*p* = 0.04)
Rice et al. 2012 [43]	Those who had moderate or major deviations from a protocol that suggested use of DC surgery when any of the following were present: temperature < 35 °C, lactate > 4 mmol/L (or more than twice the ULN), or corrected pH < 7.3 (*n* = 92)^a^	Those who had no or minor deviations from the DC surgery protocol (*n* = 358)^b^	13 variables^c^	Survival at 90 d: adjusted OR, 0.50 (95% CI, 0.27–0.92)
Martin et al. 2012 [14]	DC laparotomy for patients with an arrival systolic BP > 90 mmHg, no severe TBI (head AIS score < 3), and no combined abdominal injuries (*n* = 62)	Therapeutic definitive laparotomy in patients with an abdomen AIS score > 2	10 variables^d^	Major postoperative complication: adjusted OR, 2.96 (95% CI, 1.25–6.99) The adjusted length of hospital stay was 9.69 d longer in patients who underwent DC instead of definitive laparotomy (*p* < 0.001)
Chinnery et al. 2012 [44]	DC surgery for unstable patients with pancreatic and major associated organ and visceral vascular injuries (*n* = 43)	Before use of DC surgery for patients with this indication (*n* = 32)	None for both comparisons	Survival: OR, 0.082 (95% CI, 0.014–0.34) Postoperative complications (systemic, pancreatic, and intra-abdominal): OR, 8.02 (95% CI, 1.44–80.24)
Mayberry et al. 2011 [45]	DC laparotomy for patients with full-thickness duodenal lacerations (*n* = 25)	Definitive surgery for patients with this indication (*n* = 16)	None	Duodenal-related complications: OR, 0.38 (95% CI, 0.029-3.83)
Liu et al. 2011 [24]	DC laparotomy for patients with a preoperative temperature < 35 °C, PT > 16 s, PTT > 50 s, or pH < 7.3 or who received > 10 U PRBCs (*n* = 46)	Definitive laparotomy for patients with these indications (*n* = 58)	None for both comparisons	Survival: OR, 3.51 (95% CI, 1.18–11.73) Complications (pneumonia, peritonitis, intra-abdominal abscess, biliary or pancreatic fistula, bowel obstruction): OR, 0.29 (95% CI, 0.12–0.69)
Yu et al. 2009 [25]	DC surgery for patients with the following: preoperative temperature < 35 °C, pH < 7.25, PT > 16 s, aPTT > 50 s, or systolic BP < 70 mmHg; transfusion >10 U PRBCs; inability to close the abdomen because of visceral edema; or a predicted surgical duration > 90 min (*n* = 45)	Definitive surgery for patients with these indications (*n* = 45)	None for all comparisons	Survival: OR, 3.03 (95% CI, 0.66–18.79) Complications (abscesses, ARDS, multiple organ failure): OR, 0.29 (95% CI, 0.099–0.80) Mean ICU LOS: 10 vs. 8 d (*p* = 0.02) Mean hospital LOS: 27 vs. 21 d (*p* = 0.01)
MacKenzie et al. 2004 [51]	Laparotomy with early therapeutic perihepatic packing followed by angioembolization for patients with AAST grade IV-V liver injuries (*n* = 7)^e^	Definitive laparotomy for patients with AAST grade IV–V liver injuries (*n* = 30)^e^	None for all comparisons	Survival was 100% in the early packing versus 63.3% in the definitive laparotomy group (*p* = 0.08) Complications in the early packing vs. definitive laparotomy group included liver necrosis (OR, 4.88; 95% CI, 0.49–41.81), sepsis (OR, 1.75; 95% CI, 0.21–12.67), abscesses (OR, 6.75; 95% CI, 0.62–66.67), and bile leak (OR, 4.38; 95% CI, 0.56–35.95) Median hospital LOS: 30 vs. 10.5 d (*p* value NR) Median ICU LOS: 7 vs. 2 d (*p* value NR)
Asensio et al. 2004 [52]	After implementation of a guideline that suggested use of DC surgery for patients with the following: transfusion > 4 L PRBCs or > 5 L PRBCs and whole blood combined; total OR fluid (PRBCs and whole blood, other blood products, and crystalloid) replacement > 12 L; operating room patient temperature ≤ 34 °C, serum [HCO_3_^-^] ≤ 15 mEq/L, or arterial pH ≤ 7.2; a thoracic or abdominal vascular injury or complex hepatic injury requiring packing; those requiring ED or operating room thoracotomy; or patients that develop intraoperative coagulopathy or dysrhythmias (*n* = 53)	Before implementation of the DC surgery guideline (*n* = 86)	None for all comparisons	Survival: OR, 0.99 (95% CI, 0.42 to 2.42) Intra-abdominal abscesses: OR, 0.29 (95% CI, 0.067 to 0.95) Abdominal fistula(e): 0.34 (95% CI, 0.059 to 1.32) Extra-abdominal infection: OR, 0.34 (95% CI, 0.15 to 0.77) Abdominal wall closure: OR, 44.93 (95% CI, 11.17 to 248.12) Mean SICU LOS: 14.1 vs. 22.4 d (*p* = 0.02) Mean hospital LOS: 22.9 vs. 36.8 d (*p* = 0.08)
Apartsin et al. 2002 [23]	DC laparotomy for liver and retroperitoneal injuries (*n* = 62) or major small bowel injuries (*n* = 15)	Definitive laparotomy for liver and retroperitoneal (*n* = 59) and major small bowel injuries (*n* = 14)	None for both comparisons	Survival for liver and retroperitoneal injuries: OR, 2.73 (95% CI, 1.15 to 6.60) Survival for patients with major small bowel injuries: OR, 10.08 (95% CI, 1.44 to 80.87)
Carrillo et al. 1998 [58]	DC laparotomy for patients with penetrating injuries to the iliac vessels (*n* = 14) (11 had combined arteriovenous injuries to the common and external iliac vessels)	Definitive laparotomy for patients with this indication (*n* = 50) (13 had combined arteriovenous injuries)	None for both comparisons	Survival overall: OR, 0.71 (95% CI, 0.16–3.70) Survival for patients with combined injuries: OR, 6.25 (95% CI, 0.50–324.50)
Rotondo et al. 1993 [62]	DC laparotomy for penetrating trauma patients requiring transfusion of > 10 U PRBCs before completion or termination of laparotomy with ≥ 1 major abdominal vascular injury and ≥ 2 abdominal visceral injuries (*n* = 13)	Definitive laparotomy for penetrating trauma patients with this indication (*n* = 9)	None	Survival: OR, 26.67 (95% CI, 1.84–1296.95)
Carmona et al. 1984 [67]	Therapeutic liver packing for patients with intraoperative hemodynamic instability after more conventional techniques of hemorrhage control (e.g., direct, Pringle maneuver, hepatic artery ligation) had failed (*n* = 17)	Definitive surgery for patients with this indication who were similarly matched on age, mechanism of injury, and associated injuries (*n* = 14)	None for both comparisons	Survival: OR, 2.05 (95% CI, 0.19–27.79) Infection: OR, 0.75 (95% CI, 0.13–4.43)
Stone et al. 1983 [68]	DC laparotomy followed by closure of the abdomen under tension for patients who develop coagulopathy during operation (*n* = 17)	Definitive laparotomy for patients who develop coagulopathy during operation (*n* = 14)	None for all comparisons	Survival: OR, 23.83 (95% CI, 2.22–1102.13) All survivors (including *n* = 12 managed with DC and closure of the abdomen under tension and *n* = 1 managed with definitive laparotomy) developed complications, including wound infections (100% of those managed with DC), intra-abdominal abscesses (69.2% of the 13), and intestinal fistulae (15.4% of the 13)

Where AIS indicates Abbreviated Injury Scale; BD, base deficit; BP, blood pressure; CI, confidence interval; d, days; DC, damage control; ED, Emergency Department; GI, gastrointestinal; LOS, length of stay; MOI, mechanism of injury; NR, not reported; OR, odds ratio; PRBC, packed red blood cells; SICU, surgical intensive care unit; SSI, surgical site infection; and TBI, traumatic brain injury

^a^Variables reported to be used to generate propensity scores for matching between the groups included ISS; age; gender; mechanism of injury; ED systolic BP; ED Glasgow Coma Scale score; ED BD; ED activated clotting time; ED percent lysis at 30 min; ED PRBC transfusion; time in ED; final operating room temperature; final OR systolic BP; total operating room PRBCs; final operating room pH; final operating room BD; and final operating room lactic acid

^b^Where minor deviations included departures deemed not clinically significant; moderate deviations included care, which although departures were present, mostly followed protocol; and major deviations included those that did not meet the standards outlined in the protocol.

^c^Variables reported to be entered into the logistic regression model included age; gender; injury type; time from injury to hospitalization; PRBCs transfused before hospitalization; ISS; Glasgow Coma Scale score; shock; baseline hemoglobin, creatinine and activated PTT; country; and patients who did not require DC for both outcome comparisons

^d^Variables reported to be entered into logistic and linear regression models included age; gender; mechanism of injury; head injury; major extremity injury; combined abdominal injury; ISS; presenting vitals; BD; and need for colon resection

^e^In this study, 15 of the patients in the definitive laparotomy group were reported to ultimately need abdominal packing after conventional hepatic injury repair techniques. Moreover, 1 patient in the early therapeutic packing group received angiography before laparotomy

Of the remaining 14 studies, Chinnery et al., Rotondo et al., and Stone et al. observed a large improvement in unadjusted survival when DC or staged laparotomy was used instead of definitive surgery to manage unstable patients with combined abdominal vascular and pancreas gunshot injuries, who received > 10 U PRBCs and had ≥ 1 major abdominal vascular and ≥ 2 abdominal visceral injuries, or that developed a coagulopathy during operation, respectively [44, 62, 68]. In contrast, Harvin et al. reported that after matching injured patients on propensity scores created using 17 different variables, use of DC instead of definitive laparotomy (for intra-abdominal packing, a second-look laparotomy, hemodynamic instability, to expedite postoperative care or intervention, or for other reasons) was associated with a significantly increased incidence of gastrointestinal (GI) ileus, GI bleeding, abdominal fascial dehiscence, superficial surgical site infection, and death [38]. Moreover, Martin et al. reported that use of DC laparotomy in trauma patients with an arrival systolic BP > 90 mmHg, no severe TBI, and no combined abdominal injuries was associated with an increased adjusted odds of major postoperative complications and an increased adjusted length of hospital stay when compared to patients with a severe abdominal injury who underwent therapeutic definitive laparotomy [14].

### Narrative synthesis of validity of indications for use of DC surgery

The narrative synthesis of the aggregate evidence for use of indications for DC surgery is presented in Table 6. Of the 59 unique indications identified using directed qualitative content analysis, two had moderate or strong evidence of content validity [upper quadrant abdominal gunshot wound with a horizontal shift trajectory (e.g., from the right to the left upper quadrant) and a systolic BP < 105 mmHg or right upper quadrant wound with a bullet retained in the same quadrant and a systolic BP < 90 mmHg). Further, nine had moderate or strong evidence of construct validity (high ISS score, preoperative hypothermia, unstable patients with combined abdominal vascular and pancreas gunshot injuries, and transfusion > 10 U PRBCs and ISS score > 25 or lowest temperature < 34 °C in the pre- or intraoperative setting) and six had moderate or strong evidence of criterion validity (pre- or intraoperative hypothermia, increased lactate, or decreased pH).

**Table 6:**
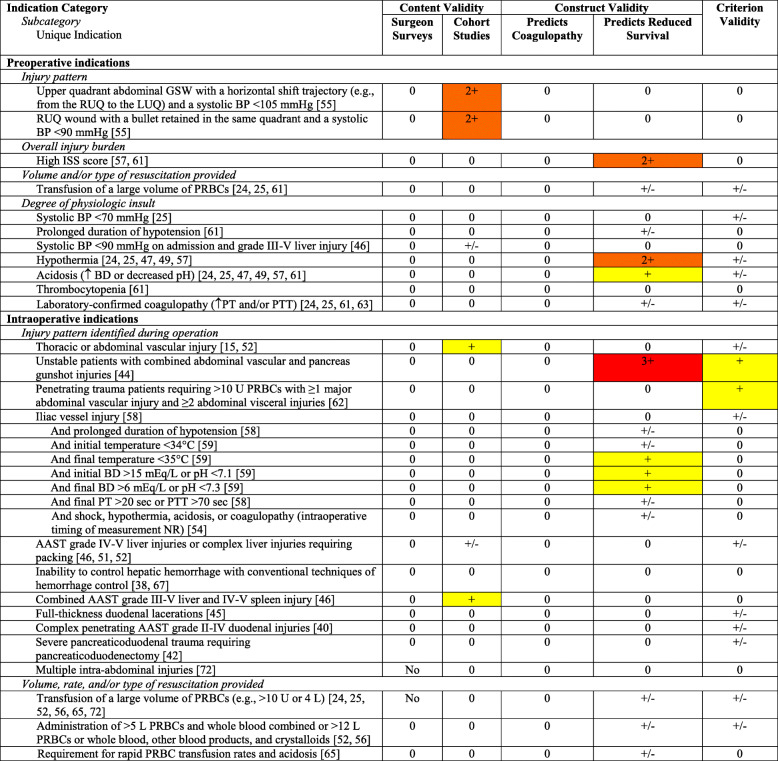

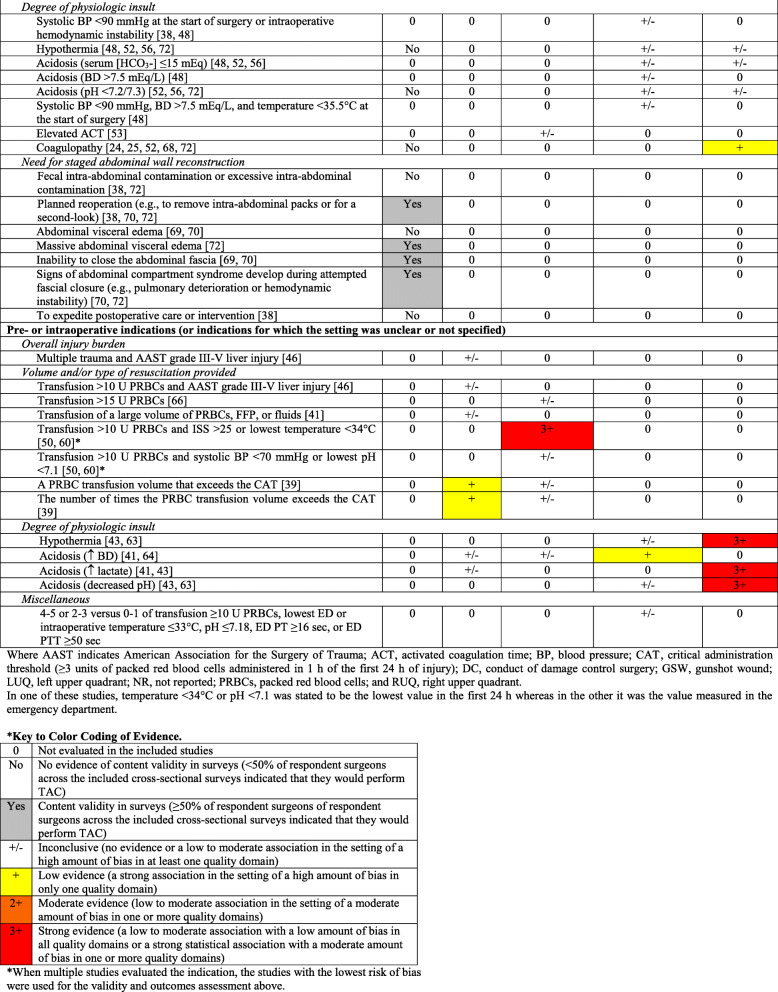
Narrative (vote counting) synthesis of evidence for indications for use of damage control surgery in civilian trauma patients

## Discussion

This systematic review is the first to comprehensively compile and critically analyze the evidence for use of DC surgery and DC interventions in civilian trauma patients. We identified 39 studies that evaluated the content, construct, and/or criterion validity of 116 indications for DC surgery. Most were single-center cohort studies that recruited relatively small samples of critically injured patients. Of the 59 unique indications identified using content analysis, 10 had evidence of content validity (i.e., surgeons self-reported that they would perform DC in that clinical scenario or the indication predicted use of DC in practice), nine had evidence of construct validity (i.e., were associated with poor outcomes in patients largely treated with definitive surgery, suggesting that DC be considered in these high-risk scenarios), and six had evidence of criterion validity (may be associated with improved outcomes when utilized or when DC was performed instead of definitive surgery).

Most included studies attempted to validate indications for use of DC surgery by assessing if they were associated with poor outcomes (i.e., coagulopathy or reduced survival); however, few studies subsequently sought to determine if DC improves survival in these situations and nearly one quarter of these studies included patients who only underwent DC (and therefore outcomes associated with these variables are better interpreted as predictors of poor outcome after DC surgery than measures of indication construct validity). Further, some physiology-based indications were associated with reduced survival in patients managed with definitive surgery [an elevated preoperative BD [57] and DC surgery [a preoperative pH < 7.20 [47]. This last finding suggests that although several measures of physiological compromise have been assessed by both international experts and practicing surgeons to be highly appropriate indications for use of DC [3, 16, 17], some data suggest that there may be a point beyond which physiologic derangements have progressed too far for DC surgery to improve survival [49].

We identified only six indications that had evidence to support that their utilization or the conduct of DC surgery may improve patient survival. These indications represent those with the most evidence to support their use and include the finding of hypothermia or acidosis, development of a coagulopathy during operation, or the identification of two injury patterns that preclude expedient definitive repair (combined abdominal vascular and pancreas gunshot injuries and ≥ 1 major abdominal vascular and ≥ 2 abdominal visceral injuries in patients who have received > 10 U PRBCs) [44, 62, 68]. However, as these were observational studies and operative profile (i.e., DC versus definitive surgery) was not randomly assigned, there were likely other, unmeasured reasons why surgeons chose to perform DC in these studies that are related to the risk of future outcomes (i.e., the studies were likely confounded by indication) [73].

Those indications with evidence suggesting that they may be associated with poor outcomes or that outcomes may be improved with use of DC surgery represent targets for focused future research efforts (Table 6). As studies cannot deliver an unbiased and meaningful assessment of validity if the type of operative procedure varies in relation to the indication of interest [28], subsequent study of indications for DC surgery must compare the outcomes of performing DC versus definitive surgery in patients with well-defined indications. This should begin with prospective cohort studies designed to estimate the causal effects of DC surgery by controlling for confounding by indication using multivariate adjustment, propensity scores, or other techniques [73]. A randomized controlled trial would provide the least biased estimates of the benefit/harm ratio of DC compared to definitive surgery in different clinical circumstances. Initial trials should randomize patients to DC or definitive surgery in those clinical circumstances with the greatest uncertainty about the potential role of DC surgery (i.e., those indications listed in Table 6 that have no or equivalent evidence of content, construct, and criterion validity). While the above studies are being designed and conducted, creation of a list of DC consensus indications may allow for the conduct of quality improvement or knowledge translation interventions to decrease overutilization of DC in trauma patients.

Collectively, the above data suggest that there exists little evidence to support the high DC surgery utilization rates reported by many level 1 trauma centers. In a recently reported post-hoc analysis of the PROPPR randomized trial, DC was used among 33% to 83% of patients requiring urgent laparotomy across 12 of the participating institutions [15]. Interestingly, although there was no significant adjusted mortality difference between these centers, the unadjusted risk of sepsis and ventilator-associated pneumonia was higher among those treated with DC laparotomy, suggesting that decreasing use of DC among individual trauma centers may not influence mortality, but may decrease associated morbidity [15]. These findings are supported by two studies included in this systematic review, which both reported that use of DC laparotomy among lower risk cohorts of injured patients was associated with increased complications and longer hospital lengths of stay [14, 38]. As these findings may have been influenced by differences in patient characteristics between groups in the above studies, they should be interpreted cautiously and confirmed by future studies.

This study has potential limitations. First, some of the indications evaluated in this systematic review were dependent on a single clinical finding. While experts and practicing surgeons have previously reported that they would conduct DC surgery when encountered with certain *single* clinical findings (e.g., massive destruction of the pancreatic head) [3, 16, 17], surgeons frequently decide to conduct DC surgery only after considering multiple clinical findings simultaneously (Table 6). Second, many of the indications assessed in the studies included in this systematic review included static physiologic or laboratory values as decision thresholds. As surveys have suggested that practicing surgeons believe that unless physiologic derangements are persistent during operation that it is likely safe to attempt a definitive trauma operation, arguably more important than any static value are the trends in these values during the early resuscitation and operative phases. Third, as our systematic review included studies of patients mostly undergoing DC surgery for torso trauma, our findings likely cannot be generalized to patients undergoing emergency general, orthopedic, or military surgery. Finally, although most of the studies included in this systematic review were reported after the year 2000, our findings must be interpreted within the context of the time range over which they were published (1983–2017). Recent changes in resuscitation practices have likely resulted in a decrease in the frequency of the need for open abdominal management because of post-injury abdominal visceral swelling [74–76]. Moreover, some have suggested that novel resuscitation strategies may potentially prevent or treat the lethal triad, which would suggest that the threshold used to select patients presenting with deranged physiology for DC surgery could potentially rise in the future pending the results of ongoing research [77].

## Conclusions

This systematic review identified a large number of indications for use of DC surgery in civilian trauma patients. Few had evidence of validity or that they were associated with improved outcomes when utilized or when DC was performed instead of definitive surgery. Appropriately designed prospective observational studies comparing the benefit-risk profile associated with conduct of DC versus definitive surgery for patients resuscitated according to currently accepted standards and treated with the indications identified in this study are therefore urgently required. In the interim, our findings support that DC should be used with respect for the uncertainty regarding its effectiveness, and only in those circumstances where definitive surgery cannot be entertained.

## Supplementary Information

**Additional file 1.** Supplemental Digital Content 1. Operationalized and Expanded QUIPS Guidelines for Assessing Risk of Bias in Included Studies. .docx file type.

**Additional file 2.** Supplemental Digital Content 2. Characteristics of the Three Cross-Sectional Studies Included in the Systematic Review. .docx file type.

**Additional file 3.** Supplemental Digital Content 3. Risk of Bias Assessment for the 35 Included Cohort Studies. .docx file type.

**Additional file 4.** Supplemental Digital Content 4. Standards for Use and Reporting of Logistic Regression in the Medical Literature.

**Additional file 5.** Supplemental Digital Content 5. Risk of Bias Assessment for the Three Included Cross-Sectional Studies.

## Data Availability

All data included and analyzed in the study have previously been published.

## Abbreviations

AAST: American Association for the Surgery of Trauma
BP: Blood pressure
CI: Confidence interval
DC: Damage control
EAST: Eastern Association for the Surgery of Trauma
ED: Emergency department
GI: Gastrointestinal
HR: Hazard ratio
ICU: Intensive care unit
ISS: Injury Severity Scale
OR: Odds ratio
PRBC: Packed red blood cells
PROPPR: Pragmatic, Randomized Optimal Platelet and Plasma Ratios
TAC: Temporary abdominal closure
WTA: Western Trauma Association
