# Supplementary material for: Evidence for use of damage control surgery and damage control interventions in civilian trauma patients: a systematic review
Source: World J Emerg Surg. 2021 Mar 11;16:10. doi: 10.1186/s13017-021-00352-5 (PMC7951941; doi:10.1186/s13017-021-00352-5)
Supplement: Supplementary file 1 — Additional file 1. Supplemental Digital Content 1. Operationalized and Expanded QUIPS Guidelines for Assessing Risk of Bias in Included Studies. .docx file type. [file 13017_2021_352_MOESM1_ESM.docx]

**Supplemental Digital Content 1. Operationalized and Expanded QUIPS Guidelines for Assessing Risk of Bias in Included Studies [23-25].**

| **Variables** | **Bias Domains** | | | | | | |
| --- | --- | --- | --- | --- | --- | --- | --- |
| **Study Quality Domains** | **Study Participation** | **Study Attrition** | **Indication Description and Measurement** | **Outcome Measurement** | **Confounding Measurement and Account** | **Similar Treatment After Cohort Inclusion** | **Statistical Analyses** |
| Prompting items | There was adequate participation in the study by eligible patients  The methods of identifying the sample and period and place of recruitment were described and appeared appropriate  For studies evaluating the validity or reliability of indications, the sample predominantly or completely included patients who underwent definitive trauma surgery  Inclusion and exclusion criteria were adequately explained  The baseline characteristics of the study sample was adequately described [e.g., types and locations of injuries, ISS scores, and presenting physiology (BD, lactate, pH, temperature, PT, PTT, INR)] | The response rate (i.e., proportion of study sample providing outcome data) was adequate  Reasons for loss to follow-up were provided  The characteristics of participants lost to follow-up were adequately described  There were likely to be no important differences between key characteristics and outcomes in participants who completed the study versus those who did not | A clear and clinically useful definition/description of the indication was provided  Continuous variables were reported or appropriate (i.e., not data-dependent) cut-offs were used  The indication was measured for all or a high proportion of the included patients | An unambiguous outcome variable was used (e.g., survival) or a clear definition of the outcome variable (e.g., DC surgery) was provided and was measured in a similar way for all patients | Important potential confounding variables, including measurements of core body temperature, acid/base status, coagulopathy and injury severity (e.g., ISS), were clearly defined and accounted for in the study design (e.g., matching, stratification, or initial assembly of comparable groups) or in the analysis  Where potential confounding variables were not accounted for in the study design or analysis, these were summarized and appeared to be balanced between the groups | Treatment (e.g., the operative profile chosen) of patients was fully described and standardized  Treatment did not depend on values of the indication variable (e.g., its presence or absence or whether its values are low or high) | There was sufficient information to assess the adequacy of the analysis or recompute the main outcome measures  If regression was appropriate, the selected model was adequate for the design of the study  If regression was appropriate, the model building strategy (i.e., inclusion of variables) was based more on theory or previous study* |
| **Ratings** | **Rating Definitions** | | | | | | |
| High risk of bias | The relationship between the indication and outcome is very likely to be different for participants and eligible nonparticipants† | The relationship between the indication and outcome is very likely to be different for completing and noncompleting participants | The measurement of the indication is very likely to be different for different levels of the outcome of interest | The measurement of the outcome had the potential for misclassification and was very likely to be different related to the baseline level of the indication | The observed “effect” of the indication on outcome is very likely to be distorted by another factor related to the indication and outcome | The treatment provided to patients is very likely to have been different for different values of the indication variable | The reported results are very likely to be spurious or biased related to analysis or reporting |
| Moderate risk of bias | The relationship between the indication and outcome may be different for participants and eligible nonparticipants | The relationship between the indication and outcome may be different for completing and noncompleting participants | The measurement of the indication may be different for different levels of the outcome of interest | The measurement of the outcome had the potential for misclassification and may be different related to the baseline level of the indication | The observed “effect” of the indication on outcome may be distorted by another factor related to the indication and outcome | The treatment provided to patients may be different for different values of the indication variable | The reported results may be spurious or biased related to analysis or reporting |
| Low risk of bias | The relationship between the indication and outcome is unlikely to be different for participants and eligible nonparticipants | The relationship between the indication and outcome is unlikely to be different for completing and noncompleting participants | The measurement of the outcome of interest is unlikely to be different for different levels of the outcome of interest | The measurement of the outcome had the potential for misclassification and is unlikely to be different related to the baseline level of the indication | The observed “effect” of the indication on outcome is unlikely to be distorted by another factor related to the indication and outcome | The treatment provided to patients is unlikely to be different for different values of the indication variable | The reported results are unlikely to be spurious or biased related to analysis or reporting |

Where BD indicates base deficit; DC, damage control; INR, international normalized ratio; ISS, Injury Severity Scale score; PT, prothrombin time; PTT, partial thromboplastin time; and QUIPS, Quality in Prognosis Studies.

*For logistic regression, the modeling strategy appeared appropriate (consider whether there were sufficient events per variable, continuous variables were reported to conform to a linear gradient, potentially important interactions were tested for, assessments were made for collinearity, and if the model was validated or goodness of fit measures were conducted) and was reported in detail (consider the selection of predictor variables, the fitting procedure, and the reporting of measures of predictive criterion validity such as whether the 95% confidence interval surrounding the point estimate of the odds ratio was provided) [27,28].

†The risk of bias was also classified as high if outcomes reported reflected estimates of prognosis after damage control surgery instead of indication validity as all of the included patients underwent damage control.
