# Supplementary material for: Evidence for use of damage control surgery and damage control interventions in civilian trauma patients: a systematic review
Source: World J Emerg Surg. 2021 Mar 11;16:10. doi: 10.1186/s13017-021-00352-5 (PMC7951941; doi:10.1186/s13017-021-00352-5)
Supplement: Supplementary file 2 — Additional file 2. Supplemental Digital Content 2. Characteristics of the Three Cross-Sectional Studies Included in the Systematic Review. .docx file type. [file 13017_2021_352_MOESM2_ESM.docx]

**Supplemental Digital Content 2. Characteristics of the Three Cross-Sectional Studies Included in the Systematic Review.**

| **Source** | **Sampling** | | **Respondent Surgeons** | | | | | |
| --- | --- | --- | --- | --- | --- | --- | --- | --- |
|  | **Method** | **Frame** | **No.** | **RR (%)** | **Practice Location (%)** | **% Academic Practice Setting** | **Median Yrs. in Practice** | **Fellowship Training (%)** |
| MacLean *et al*., 2008 [[66]](#_ENREF_66) | Purposive | AAST members with a valid e-mail address | 103 | 26 | U.S.A. (100) | 73 | NR | Trauma and critical care (45), critical care (13), vascular surgery (3), other (8) |
| Kirkpatrick *et al*., 2006 [67] and Karmali *et al*., 2006 [68] | Purposive | Trauma Association of Canada members whose reported specialty was general, trauma, pediatric, or vascular surgery | 86 | 84 | Canada (100) | NR | 7 | NR |
| Mayberry *et al*., 1999 [69] | Purposive | 473 AAST members | 292 | 70 | U.S.A. (100) | 85 | NR | NR |

Where AAST indicates American Association for the Surgery of Trauma; NR, not reported; and RR, response rate.
