# Supplementary material for: Evidence for use of damage control surgery and damage control interventions in civilian trauma patients: a systematic review
Source: World J Emerg Surg. 2021 Mar 11;16:10. doi: 10.1186/s13017-021-00352-5 (PMC7951941; doi:10.1186/s13017-021-00352-5)
Supplement: Supplementary file 3 — Additional file 3. Supplemental Digital Content 3. Risk of Bias Assessment for the 35 Included Cohort Studies. .docx file type. [file 13017_2021_352_MOESM3_ESM.docx]

**Supplemental Digital Content 3. Risk of Bias Assessment for the 35 Included Cohort Studies.**

| **Source** | **Risk of Bias Rating*** | | | | | | |
| --- | --- | --- | --- | --- | --- | --- | --- |
|  | **Study Participation** | **Study Attrition** | **Indication Description/Measurement** | **Outcome Measurement** | **Confounding Measurement/Account** | **Similar Treatment After Cohort Inclusion** | **Statistical Analyses** |
| Watson *et al*., 2017 [[15]](#_ENREF_15) | Low | Low | Low | Low | Moderate | Low | Moderate |
| Harvin *et al*., 2016 [[35]](#_ENREF_35) | Low | Low | Moderate | Moderate | Low | Low | Low |
| Savage *et al*., 2014 [[36]](#_ENREF_36) | Low | Low | Low | Moderate for DCL/low for survival | High | Low for DCL/  high for survival | Low |
| Ordoñez *et al*., 2014 [[37]](#_ENREF_37) | High | Moderate | Moderate | Low | High | NA | Low |
| Mahmood *et al*., 2014 [[38]](#_ENREF_38) | Low | Low | Moderate | Moderate | Moderate | Low | Moderate |
| Thompson *et al*., 2013 [[39]](#_ENREF_39) | Low | Low | Moderate | Low | High | NA | Low |
| Rice *et al*., 2012 [[40]](#_ENREF_40) | Low | Low | Low | Low | Low | NA | Low |
| Martin *et al*., 2012 [[14]](#_ENREF_14) | Low | Low | Low | Moderate | Low | Low | Moderate |
| Chinnery *et al*., 2012 [[41]](#_ENREF_41) | Low | Low | Moderate | Low | Moderate for the reported indication/high for survival | Moderate for indication validity/NA for outcomes associated with DCL | Moderate |
| Mayberry *et al*., 2011 [[42]](#_ENREF_42) | Low | Low | High | Low | High | NA | Low |
| Liu *et al*., 2011 [[21]](#_ENREF_21) | Low | Low | Low | Low | High | NA | Low |
| Leppäniemi *et al*., 2011 [[43]](#_ENREF_43) | Low | Low | Low | Low | Unsure | Moderate | Moderate |
| Timmermans *et al*., 2010 [[44]](#_ENREF_44) | High† | Low | Low | Low | Moderate | Low | Moderate |
| Matsumoto *et al*., 2010 [[45]](#_ENREF_45) | High† | Low | Low | Low | High | Low | Low |
| Kairinos *et al*., 2010 [[46]](#_ENREF_46) | Low | Low | Low | Low | Moderate | Low | Moderate |
| Yu *et al*., 2009 [[22]](#_ENREF_22) | Moderate | Low | Low | Low | High | NA | Low |
| Kashuk *et al*., 2008 [[47]](#_ENREF_47) | Low | Low | Low | Low | Low | Unsure | Moderate |
| MacKenzie *et al*., 2004 [[48]](#_ENREF_48) | Moderate | Low | Moderate | Low | High | NA | Low |
| Asensio *et al*., 2004 [[49]](#_ENREF_49) | Low | Low | Moderate | Low for survival/moderate for others | High | NA | Low |
| Aucar *et al*., 2003 [[50]](#_ENREF_50) | High | Low | Moderate | High | Unsure | Low | Moderate |
| Asensio *et al*., 2003 [[51]](#_ENREF_51) | Low | Low | High | Low | Moderate | Moderate | High |
| Hirshberg *et al*., 2002 [[52]](#_ENREF_52) | Moderate | Low | Low | Moderate | Moderate | Low | Low |
| Apartsin *et al*., 2002 [[20]](#_ENREF_20) | High | Unsure | Low | Low | Unsure | NA | High |
| Asensio *et al*., 2001 [[53]](#_ENREF_53) | High† | Low | Low | Low | Moderate for transfusion >4 L PRBCs/high for other indications | Low | Moderate |
| Krishna *et al*., 1998 [[53]](#_ENREF_54) | Low | Low | Low | Low | Moderate | Low | Low |
| Carrillo *et al*., 1998 [[55]](#_ENREF_55) | Low | Low | Low | Low | High | Low | Moderate |
| Cushman *et al*., 1997 [[56]](#_ENREF_56) | Low | Low | Low | Low | High | Moderate | Low |
| Cosgriff *et al*., 1997 [[57]](#_ENREF_57) | Low | Low | Low | Low | Low | Low | Moderate |
| Garrison *et al*., 1996 [[58]](#_ENREF_58) | High† | Low | Moderate | Low | High | Low | Low |
| Rotondo *et al*., 1993 [[59]](#_ENREF_59) | Low | Low | Moderate | Low | High | NA | Low |
| Sharp and Locicero, 1992 [[60]](#_ENREF_60) | High† | Low | Low | Low | High | Low | Low |
| Rutherford *et al*., 1992 [[61]](#_ENREF_61) | Moderate | Low | Low | Low | Moderate | Unsure | Moderate |
| Burch *et al*., 1992 [62] | Low | Low | Low | Low | Moderate | Low | Moderate |
| Cué *et al*., 1990 [[63]](#_ENREF_63) | High† | Low | Moderate | Low | High | Moderate | Low |
| Carmona *et al*., 1984 [[64]](#_ENREF_64) | Low | Low | Moderate | Low | High | NA | Low |
| Stone *et al*., 1983 [[65]](#_ENREF_65) | Low | Low | Moderate | Low | High | NA | Low |

DCL, damage control laparotomy; LOS, length of stay; and NA, not applicable (as outcomes were compared between groups that were managed with damage control (DC) versus definitive surgery for one or more indications).

*Criteria used to determine whether individual study quality domains were rated as having a low, moderate, or high risk of bias are described in the Table in **Supplemental Digital File 2**.

†As these studies included a high proportion (or only) patients who underwent damage control, their reported estimates relate more to prognosis after damage control than indication validity.
