# Supplementary material for: Evidence for use of damage control surgery and damage control interventions in civilian trauma patients: a systematic review
Source: World J Emerg Surg. 2021 Mar 11;16:10. doi: 10.1186/s13017-021-00352-5 (PMC7951941; doi:10.1186/s13017-021-00352-5)
Supplement: Supplementary file 4 — Additional file 4. Supplemental Digital Content 4. Standards for Use and Reporting of Logistic Regression in the Medical Literature. [file 13017_2021_352_MOESM4_ESM.docx]

**Supplemental Digital Content 4. Standards for Use and Reporting of Logistic Regression in the Medical Literature [27].**

| **Source** | **Events per Variable** | **Conformity with Linear Gradient*** | **Interactions** | **Collinearity** | **Predictor Variable Statistics** | **Variable Selection** | **Fitting Procedure** |
| --- | --- | --- | --- | --- | --- | --- | --- |
| Watson *et al*., 2017 [[15]](#_ENREF_15) | Use of DC: 329/4=82.3 | NR | NR | Pearson’s correlation coefficient and examination of the variance inflation factor | CI, p-value | NR | Unclear |
| Harvin *et al*., 2016 [[35]](#_ENREF_35) | NA | NR | NR | NR | CI, p-value | Variables available to the surgeon before the decision to conduct DC laparotomy | NR |
| Mahmood *et al*., 2014 [[38]](#_ENREF_38) | 15/5=3 | NR | NR | NR | CI, p-value | NR | NR |
| Rice *et al*., 2012 [[40]](#_ENREF_40) | 30 d mortality: 48/14=3.4  90 d mortality: 54/14=3.9 | NR | NR | NR | CI, p-value | Previously published associations with mortality in trauma patients and authors’ clinical judgment | NR |
| Martin *et al*., 2012 [[14]](#_ENREF_14) | Major postoperative complication: 264/10=26 | NR | NR | NR | CI, p-value | Variables not associated with poor outcomes in bivariate analyses | NR |
| Chinnery *et al*., 2012 [[41]](#_ENREF_41) | Mortality: 46/12=3.8  Complicati-ons: 150/11=13.6 | NR | NR | NR | CI, p-value | Bivariate, p<0.05 | NR |
| Leppäniemi *et al*., 2011 [[43]](#_ENREF_43) | Unclear | NR | NR | NR | NR | NR | Forward stepwise |
| Timmermans *et al*., 2010 [[44]](#_ENREF_44) | 20/4=5 | NR | NR | NR | p-value | Bivariate, p<0.05 | NR |
| Kashuk *et al*., 2008 [[47]](#_ENREF_47) | Unclear | Yes | NR | NR | Coefficient, CI, p-value | Risk factors for coagulopathy identified from previous study[^57^](#_ENREF_57) | Unclear |
| Aucar *et al*., 2003 [[50]](#_ENREF_50) | Unclear | NR | NR | NR | None | Unclear | Unclear |
| Asensio *et al*., 2003 [[51]](#_ENREF_51) | Unclear | NR | NR | NR | CI, p-value | Bivariate that did not have more than 10% missing data, p<0.20 | Stepwise |
| Asensio *et al*., 2001 [[53]](#_ENREF_53) | Unclear | NA | NR | NR | CI, p-value for unadjusted estimates only | Bivariate, p<0.20 | NR |
| Krishna *et al*., 1998 [[54]](#_ENREF_54) | 13/3=4.3 | NR | NR | NR | CI | Informal† | Forward stepwise |
| Cosgriff *et al*., 1997 [[57]](#_ENREF_57) | 27/5=5.4 | NR | NR | NR | Coefficient, CI, p-value | Bivariate, p<0.25 | NR |
| Garrison *et al*., 1996 [[58]](#_ENREF_58) | NA | NA | NA | NA | NA | NA | NA |
| Rutherford *et al*., 1992 [[61]](#_ENREF_61) | 458/6=76.3 | NR | BD*TBI, BD*penetrating MOI, BD*age ≥55 years | NR | CI, p-value | In alignment with those used in TRISS | Forward stepwise |
| Burch *et al*., 1992 [[62]](#_ENREF_62) | Unclear | NR | NR | NR | p-value | Unclear | Unclear |

Where BD indicates base deficit; CI, confidence interval; DC, damage control; MOI, mechanism of injury; NA, not applicable; NR, not reported; TBI, traumatic brain injury (defined as an Abbreviated Injury Scale score ≥3); and TRISS, Trauma and Injury Severity Score.

*For continuous predictor variables.

†Although the selection of variables for inclusion in the model was not data driven, the rationale for their inclusion was not described.
