# Supplementary material for: Evidence for use of damage control surgery and damage control interventions in civilian trauma patients: a systematic review
Source: World J Emerg Surg. 2021 Mar 11;16:10. doi: 10.1186/s13017-021-00352-5 (PMC7951941; doi:10.1186/s13017-021-00352-5)
Supplement: Supplementary file 5 — Additional file 5. Supplemental Digital Content 5. Risk of Bias Assessment for the Three Included Cross-Sectional Studies. [file 13017_2021_352_MOESM5_ESM.docx]

**Supplemental Digital Content 5. Risk of Bias Assessment for the Three Included Cross-Sectional Studies.**

| **Source** | **Methods Described Permit Replication** | **Sample Likely Representative of Population** | **Questionnaire** | | | | **Statistical Methods Clearly Reported and Appropriate** | **All Respondents Accounted for** |
| --- | --- | --- | --- | --- | --- | --- | --- | --- |
|  |  |  | **Adequately Described** | **Pretested** | **Evidence of Reliability** | **Evidence of Validity** |  |  |
| MacLean *et al*., 2008 [[66]](#_ENREF_66) | No | No | No | Unclear | No | No | Yes | Yes |
| Kirkpatrick *et al*., 2006 [[67]](#_ENREF_67) and Karmali *et al*., 2006 [[68]](#_ENREF_68) | Yes | Yes | Yes | Yes | No | No | Yes | Yes |
| Mayberry *et al*., 1999 [[69]](#_ENREF_69) | Yes | Yes | Yes | Unclear | No | No | Yes | Yes |
